# Analogous Case of Conversion of Disease

**Published:** 1817-08

**Authors:** 


The following Outline of a Case, somewhat analogous to the foregoing, has been furnished us by an ingenious and intelligent Physician of our Acquaintance. EDITORS.

---

" EARLY in the year 1814, I was requested to visit Mr. ——, a gentleman about 60 years of age, of inactive habits, corpulent, with a particularly ruddy complexion, and a hearty eater of good things. I learnt, from the intelligent general practitioner in attendance, that Mr. —— had, for many weeks, suffered from a train of anomalous symptoms, giving different indications at different periods; sometimes leading to a supposition that the heart might be morbidly affected; at others, that the encephalon was the seat of disease. In communication with the patient, I was informed, that he chiefly suffered from vertigo, preceded by palpitation of the heart and ' distressing feel about the chest.' The bowels, which had been habitually very lax, were now torpid; the pulse irregular and slow, seldom reaching to 68 pulsations, oftener only 60; appetite good; no particular thirst; tongue white. I do not recollect precisely what remedial means were directed; only, that for several days following he complained of greater distress, the pulse becoming slower, even to 48, when æther and volatiles were employed, with such marked advantage, that no other medicines were directed, and the patient got so well in about a fortnight that my attendance was dispensed with. Some little time afterward, the same train of symptoms recurred, and shortly yielded to antispasmodics, except a vertiginous feel, for which a seton in the neck was adopted, and a regular use of eccoprotics directed. These means were continued for nearly four months with progressive advantageous effect, when my presence was again requested. Mr. —— was now free from all his former complaints, but was greatly alarmed at what he called 'a swelled testicle:' this, on examination, the attendant surgeon and myself pronounced *hydrocele.* Our patient would not consent to any operation; and we were dismissed, with a promise of hearing from him again if he got worse. Within three months from this, the hydrocele suddenly disappeared; a few days afterwards the vertiginous affection (from which he had been completely free during the existence of hydrocele) returned in a slight degree, and so continued, with little variation, for several weeks, when, while at breakfast, he suddenly fell from his chair in a strongly marked apoplectic state, and, notwithstanding the prompt use of remedial means, expired in ten hours. L."

